# Novel Interlinked Suspensory Fixation Technique Demonstrates Excellent Biomechanics and Improves Patient Outcomes in Anterior Cruciate Ligament (ACL) Reconstruction With Quadriceps Tendon Autograft

**DOI:** 10.7759/cureus.107478

**Published:** 2026-04-21

**Authors:** Patrick Garvin, Jensen Kolaczko, Lauren M Heylmun, Brianna Rowe, Trevor J Wait, Emily Glynn, Eric Griffith, James Genuario

**Affiliations:** 1 Orthopedics, University of Colorado Anschutz Medical Campus, Aurora, USA; 2 Orthopedics and Sports Medicine, UCHealth Steadman Hawkins Clinic, Englewood, USA; 3 Orthopaedic Surgery, Orthopedics, and Sports Medicine, UCHealth Steadman Hawkins Clinic, Englewood, USA

**Keywords:** adjustable-loop fixation, anterior cruciate ligament reconstruction, biomechanical testing, patient-reported outcome measures, surgical technique

## Abstract

Introduction

Quadriceps tendon (QT) autografts are a versatile option for primary anterior cruciate ligament reconstruction (ACLR) due to low donor site morbidity, favorable biomechanical properties, and reliable clinical outcomes. Suspensory loop fixation techniques have been described for QT graft preparation; however, concerns regarding graft strength and construct stability persist. This study aims to evaluate the biomechanical strength and report clinical outcomes of a novel interlinked adjustable loop suspensory (IAL) fixation technique for all-soft tissue QT autograft preparation for ACLR.

Methods

This study combined controlled laboratory biomechanical testing with retrospective clinical case series. Strengthening the Reporting of Observation Studies in Epidemiology (STROBE) guidelines for cohort studies were followed. QT tendon grafts were harvested from fresh-frozen cadaveric knee specimens and trimmed to a diameter of 9.5 mm and length of 75 mm. Grafts were prepared following the IAL technique. All grafts underwent cyclic loading of 50-250 N for 2,000 cycles at 1 Hz, followed by ultimate load to failure testing (ULTF) at a displacement rate of 0.33 mm/s. Elongation under cyclic loading, ULTF, and mode of failure were recorded. Clinical data were collected for patients undergoing primary ACLR with this graft preparation, with or without concomitant meniscus treatment. Patient-reported outcome measures (PROMs) and range of motion (ROM) data were retrospectively collected from the medical record.

Results

Five QT grafts underwent biomechanical testing. The mean elongation was 4.16±0.42 mm over 2000 cycles, with minimal additional elongation between cycles 1000 and 2000 (0.09±0.01 mm). Mean ULTF was 1251.41±134.78 N. Graft failure occurred at the suture-tendon interface, with two grafts breaking at the loop and three demonstrating suture tail slippages. Thirty-three skeletally mature patients were included in this study. All patients demonstrated significant and clinically meaningful improvements in pain and functional outcomes at two-year follow up, and flexion and extension ROM at the six-month follow-up. Two (6.1%) patients had recurrent ipsilateral ACL tears due to re-injury; none of the patients experienced graft failure.

Conclusion

This novel interlinked adjustable-loop QT graft preparation demonstrates promising biomechanical characteristics and satisfactory early clinical outcomes, supporting its use as an effective preparation technique for all-soft tissue QT autografts in ACLR.

## Introduction

Anterior cruciate ligament (ACL) tears are among the most common orthopedic injuries, affecting approximately one in 1,500 people annually, most often during athletic activity [1,2]. ACL injury rates vary by activity type and sex, where women participating in contact and high-impact rotational landing sports are at higher risk [3]. ACL reconstruction (ACLR) aims to restore knee stability and function by replacing the torn ACL with a graft that exhibits similar structure and biomechanics. Graft choices for ACLR include both allografts and autografts where hamstring tendon (HT), quadriceps tendon (QT) and patellar tendon (PT) are commonly used. Graft selection depends on several key factors, including patient age, activity level and type, and barriers to rehabilitation where PT and QT autografts are preferred for younger, more active patients [4].

Clinical failure and graft re-ruptures affect an estimated 5% and 20% of all primary ACLR cases, respectively [5]. Risk factors for re-rupture and revision ACLR include male sex, younger age, returning to high level of activity, femoral tunnel placement, and graft selection, with allograft and HT autograft associated with increased risk of failure [6]. While there is no universally ideal graft choice, preference for QT autograft has been increasing due to its favorable biomechanics and clinical outcomes [7]. Compared to HT and PT autografts, QT autografts demonstrate larger cross-sectional area, which may contribute to enhanced strength and stability [8-10]. Additionally, the ultimate load of QT autografts exceeds HT autografts with some studies demonstrating mixed but generally comparable results between QT and PT autografts harvested with bone block [9-11]. Clinical outcomes following ACLR with QT autograft are similar to PT with both QT and PT demonstrating superior outcomes compared to HT [10, 12, 13]. Patients with QT autograft additionally report lower donor-site morbidity and demonstrate greater post-surgical extension strength on the donor limb compared to PT autograft with bone block [10, 12].

During ACLR, securing the graft to the bone may be achieved with aperture fixation, where the graft is secured within the bone tunnel on the proximal femur or distal tibia, or with suspensory fixation, where a button secures the graft at a distance from the cortical surface [14]. Both techniques demonstrate comparable ultimate load to failure and stiffness, although aperture fixation has been associated with less displacement under cyclic loading [15]. Clinically, both fixation methods improve joint stability with similar outcomes [16, 17]. Biomechanics studies comparing continuous and fixed-loop suspensory fixation techniques report mixed findings [17-20] with variability likely related to differences in suturing technique, number of throws, and suture diameter [21]. Notably, the predominant mechanism of failure across these constructs was suture pull-through or soft tissue shredding at the fixation site, highlighting a persistent limitation of single-point fixation at the tendon-suture interface [18,19,22].

Given this limitation, there remains a need for fixation strategies that reduce stress concentration at the suture-tendon interface and improve load distribution across the graft. This study introduces a novel suspensory fixation technique using interlinked adjustable loops (IAL) for QT autograft preparation in ACLR. By interlinking continuous loops on the femoral and tibial sides, this construct is designed to distribute load more evenly throughout the graft and mitigate failure at the suture-tendon interface. The objectives of this study are to (a) describe the IAL technique, (b) evaluate its biomechanical properties, and (c) report early clinical outcomes in patients undergoing ACLR with this construct.

## Materials and methods

All soft-tissue QT grafts were harvested from five fresh-frozen cadaveric knee specimens with a minimum of 10 mm diameter and 75 mm length. Demographic information for cadaveric specimens was not available. All grafts were trimmed to a uniform 9.5 mm diameter and 75 mm length. Two post-graduate year 6 (PGY6) orthopaedic sports medicine fellows with a certified first assistant prepared the grafts under supervision of a board-certified orthopedic sports medicine surgeon. Grafts were thawed overnight and allowed to cool down to room temperature prior to graft preparation and testing.

Specimen preparation and repair technique

All grafts were harvested via a longitudinal incision made with a 15-blade extending approximately 8-10 cm from the superior pole of the patella to aid in exposure. The graft was elevated full thickness off the underlying knee capsule. Graft harvest began proximally with dissecting scissors and 15 blades, taking care to preserve the 10-mm graft. After adequate exposure of at least 75-80 mm of graft, a cut at the proximal extent was made to complete the harvest. Graft preparation for the IAL construct employed a novel interlinked adjustable loop (ProCinch reverse tensioning and ProCinch Open Loop, Stryker Endoscopy, San Jose, CA) suspensory fixation technique.

**Figure 1:**
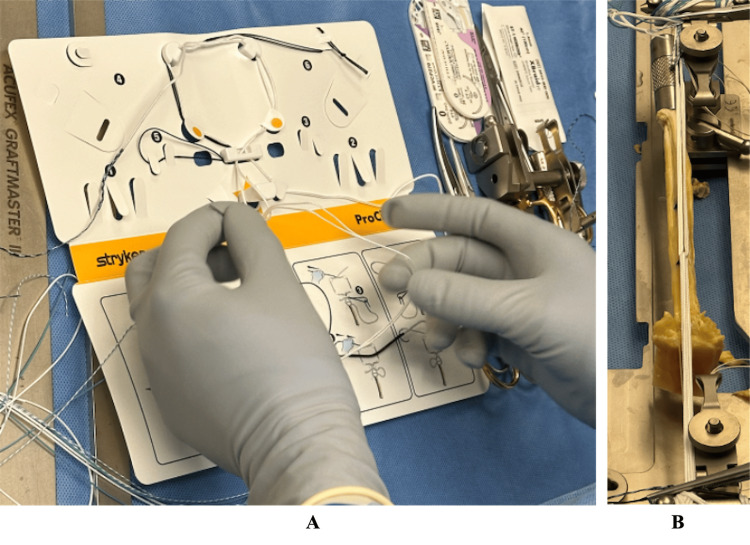
Interlinked Femoral and Tibial Suspensory Loop Preparation Interlinked femoral and tibial suspensory loop preparation. Left image (A) shows suture construct being prepared with graft preparation board (Acufex GraftMaster, Smith & Nephew). Right image (B) visualizes quadriceps tendon (QT) graft with incorporated suture fixation ProCinch RT and ProCinch Open Loop, Stryker Endoscopy).

This was completed by interlinking two adjustable suspensory loops, the reverse tensioning (RT) for the femoral side and the open loop (OL) on the tibial side. The OL was shuttled through the RT closed adjustable loop using the provided preparation board (Figure 1A). The lengths of each loop were equal to allow for tensioning and to form the link later attached to the tendon.

**Figure 2:**
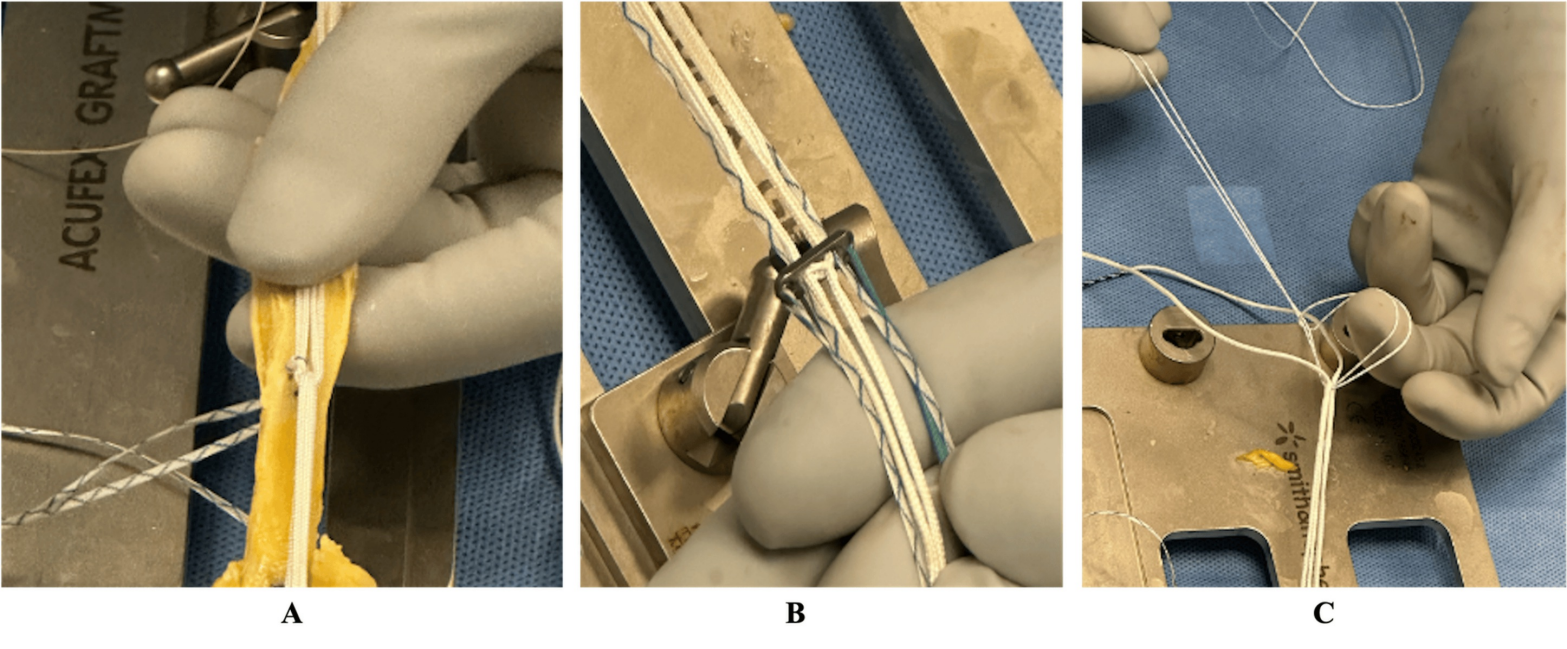
Interlinked Suspensory Loop (IAL) Preparation Technique (A) Reverse tensioning suspensory cortical button fixation (Procinch reverse tensioning, Stryker Endoscopy) on femoral side of quadriceps tendon (QT) autograft. (B) Visualization of the tape limbs (Xbraid TT, Stryker Endoscopy) through the central holes of the button to be used for graft tensioning. Additional suture looped to act as a luggage tag pull on the tibial side of the graft.

Using a graft preparation board (Acufex GraftMaster, Smith & Nephew, Memphis, TN), the quadriceps tendon was secured between two clamps, with the center of the graft aligned with the center of the interlinked suture construct (Figure 1B). Starting with the femoral side of the graft, an additional 1.4-mm high tensile strength suture tape (XBraid TT, Stryker) was passed through the graft and the suture loop with a Keith needle, exiting either side of the loop using non-locking whip stitch configuration to secure suture construct to the graft. After the final pass, the suture was brought through the tendon and through the loop of the previous pass to create a “rip stop” and lock the suture (Figure 2A). Care was taken to avoid piercing the suture of the link itself, with each pass placed adjacent to the loop. Caution was critical, as inadvertent piercing of the loop suture can preclude tension of the construct.

**Figure 3:**
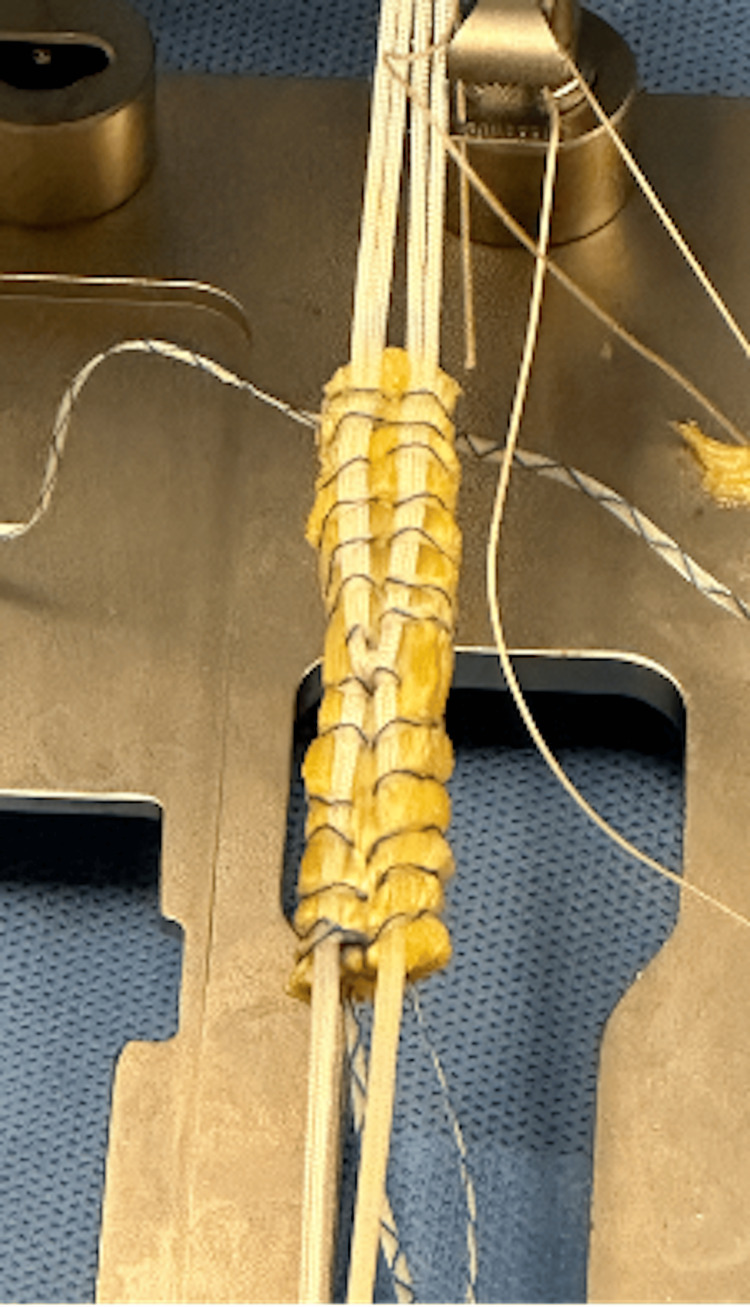
Final Quadriceps Tendon Autograft Construct Final quadriceps tendon (QT) autograft construct prepared with suspensory interlinked loop fixation (IAL) technique.

This process was then repeated with an additional 1.4-mm high tensile strength suture tape to secure the loop to the tibial fixation side of the graft. Once the graft was secured to the interlinked suspensory loop, the femoral side suture limbs were passed through the additional holes in the RT suspensory cortical button (Figure 2B). On the tibial side, an additional suture was placed in luggage tag configuration to act as a pull-stitch to aid in graft passage and to avoid inadvertent shortening of the limbs (Figure 2C). The final construct is visualized in Figure 3.

Biomechanics, technical setup, and data collection

**Figure 4:**
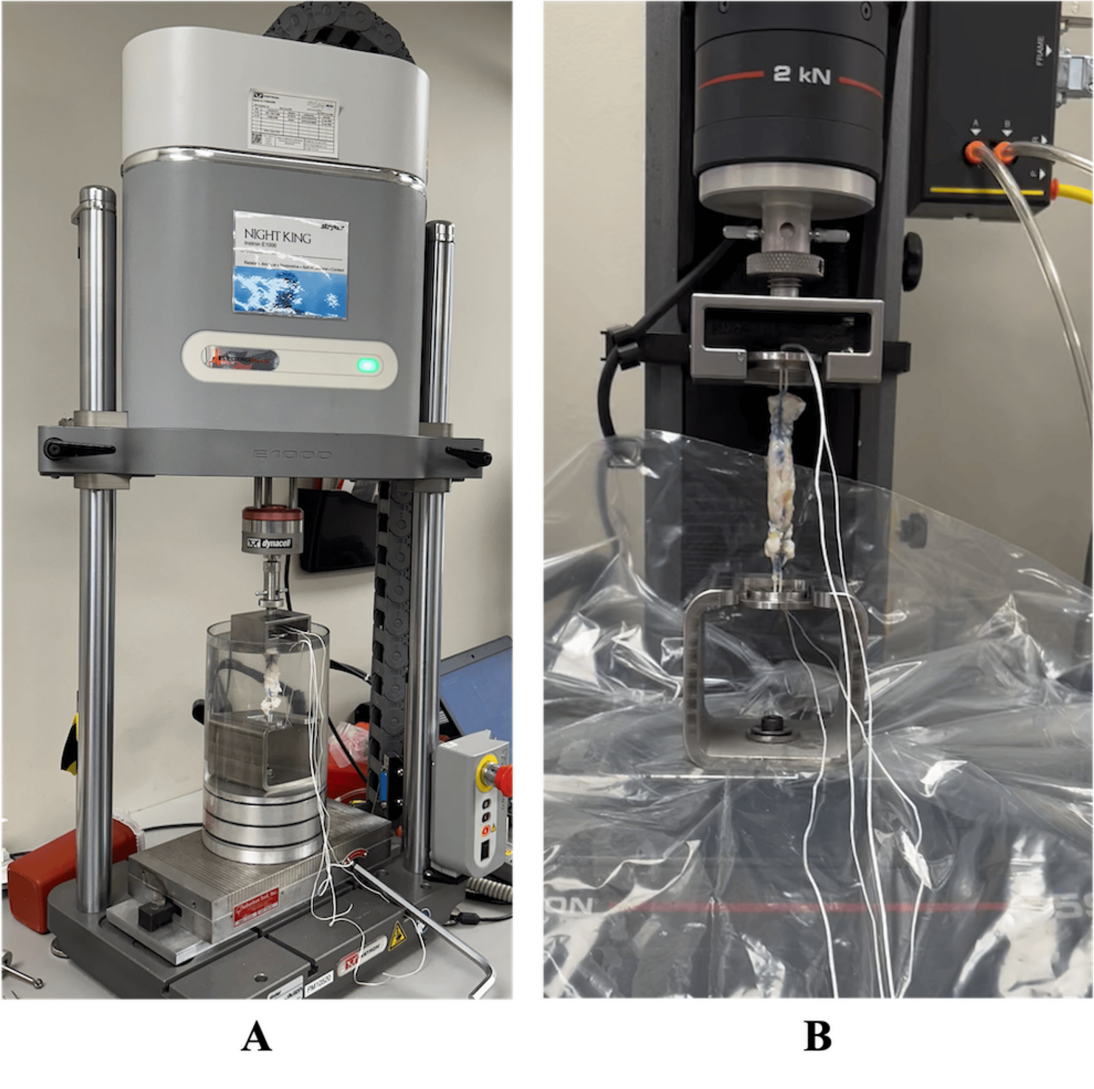
Biomechanic Testing Setup (A) Visualization of cyclic load testing setup using Mark-10 EM ESM301 test stand with M5-200 force gauge. (B) Visualization of ultimate load to failure testing setup using INSTRON system.

The loops on either end of the graft were secured and tensioned over a metal washer using a metal pin. The grafts were submerged in room-temperature distilled water and secured to the ESM301 Test Stand (Mark-10, Copiague, NY) by sliding the washers into the upper and lower recessed cavities of the stand (Figure 4A). The test stand was equipped with an M6-200 Force gauge and was preloaded to 50 N. For cyclic load testing, mechanical forces alternated between 50 and 250 N at a frequency of 1 Hz for 2000 cycles, reflecting physiologic loading conditions during activities such as walking. Graft elongation (mm) was calculated as the change in length between the initial extension under a 50 N preload and the final extension after 2000 loading cycles. The selected cycle count was intended to simulate repetitive loading encountered during early postoperative activities. Graft elongation between 1000 and 2000 cycles was measured in the same fashion.

Subsequently, the grafts were transferred to ElectroPlus E1000 System or INSTRON 5944 System (INSTRON, Norwood, MA) to determine the ultimate load to failure (UTLF) (N). Grafts were loaded onto the machine by inserting the metal washers into the recessed cavity of the machine stand (Figure 4B). A uniaxial tensile load was applied at a constant displacement rate of 0.33 mm/s (20 mm/min), consistent with prior biomechanical testing protocols, until failure. The mode of failure for each specimen was documented.

Case series

Following institutional review board (IRB) approval, patients who underwent primary ACLR with QT autograft prepared following the IAL technique at a single institution between December 2021 and October 2023 were retrospectively reviewed. Skeletally mature patients with and without meniscus treatments and a minimum of two years of follow-up data were included. Exclusion criteria consisted of revision cases, contralateral surgery performed within one year of initial surgery, concomitant ligamentous, and missing patient-reported outcome measure (PROM) data at three or more timepoints.

Patient demographics, condition history, surgical treatment, and PROMs were recorded prospectively at the time of treatment and retrospectively collected for this study. All patients followed the institution's standard postoperative ACLR rehabilitation protocol. Post-surgical complications and revision surgeries related to the index knee were recorded. PROMs included Patient Reported Outcomes Measurement Information System (PROMIS, HealthMeasures, Evanston, IL) pain interference (PROMIS PI), PROMIS physical function (PROMIS PF), and Knee Injury and Osteoarthritis Index Score (KOOS, Lund University) activities of daily living (ADL) and sport sub-scales [23,24]. PROMs were measured at baseline and three, six, 12 and 24 months postoperatively. Knee flexion and extension ROM were measured by a licensed clinician at baseline, six weeks, 12 weeks, 18 weeks, and six months postoperatively.

Statistical analysis

All statistical analyses were performed using Python (version 3.11.9, Python Software Foundation, Wilmington, DE, https://www.python.org/). Descriptive statistics are reported as mean±standard deviation (SD) for continuous variables, and frequency and proportion (%) for categorical variables. Linear mixed models (LMM) were used to analyze longitudinal trends in PROMs and ROM. This method was chosen to appropriately account for within-subject correlation arising from repeated measures over time and to allow inclusion of all available observations without requiring complete data at every time point. Adjustment for multiple comparisons was not performed, as the primary analysis was based on a pre-specified longitudinal linear mixed-effects model evaluating changes in outcomes over time. Missing data were not imputed and LMMs were fit using restricted maximum likelihood (REML) estimation, which incorporates all available data under the assumption of missing at random. LMMs included time as a fixed effect and patient ID as a random effect to account for repeated measures within individuals. Patients were stratified by sex and presence of meniscus treatment for secondary analysis. The presence of meniscus treatment was grouped as a binary variable to indicate the presence of meniscus treatment vs no meniscus treatment. Statistical significance was set at α=0.05.

## Results

Biomechanical testing results

Five QT grafts were harvested from five fresh-frozen cadavers. Demographic information for the cadavers was unavailable. All grafts were trimmed to 9.5 mm diameter and 75 mm length. All five grafts completed both phases of biomechanical testing, including cyclic loading and ultimate load to failure. Test results for individual grafts and means are summarized in Table 1.

**Table 1:** Cyclic Loading and Ultimate Load to Failure (ULTF) Testing Results

Specimen No.	Graft Elongation (mm)		ULTF (N)
	1-2000 cycles	1000-2000 cycles	
1	4.07	0.1	1235.58
2	3.65	0.09	1449.84
3	4.81	0.1	1157.76
4	4.21	0.08	1106.17
5	4.05	0.07	1307.72
Mean	4.16	0.09	1251.41
Standard Deviation	0.42	0.01	134.8

**Figure 5:**
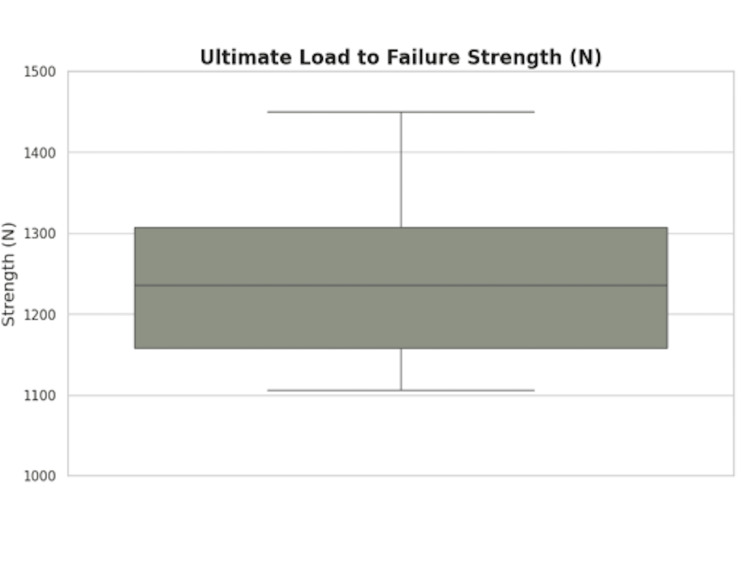
Biomechanic Testing Results Boxplot (left) visualizing distribution of graft elongation (mm) measurements following 1-2000 cycles and 1000-2000 cycles. Boxplot (right) visualizing distribution of ultimate load to failure (ULTF) (N) measurements.

The mean total elongation following 2000 cycles of cyclic loading was 4.16±0.42 mm, with a mean elongation of 0.09±0.01 mm observed between 1000 and 2000 cycles (Figure 5). The mean ULTF was 1251.4±134.8 N (Figure 5).

Graft failure at ultimate load occurred due to slippage of the fixation loop in three grafts (60%) and suture loop breakage in two grafts (40%). Notably, no failures were observed at the suture-tendon interface.

Retrospective case series

Thirty-three patients with primary full-thickness ACL tears who underwent ACLR with the IAL construct QT autograft were included in this retrospective case series. Patient demographics are reported in Table 2. Two (6.1%) patients underwent revision ACLR following secondary injuries sustained more than one year after the index procedure; for these patients, outcome data collected after revision surgery were excluded from the analysis to maintain consistency with the index procedure cohort. Four (12.1%) patients required secondary procedures for anterior interval scarring, and 1 (3.0%) underwent secondary surgery for tibial button removal. Postoperative infection of the index knee occurred in two patients: one secondarily to an infection that developed following an unrelated procedure and the second after a traumatic fall that reopened and exposed the surgical incision. No cases of graft failure were observed.

**Table 2:** Patient Demographics ACL: Anterior cruciate ligament.

All patients (n=33)		Mean± SD
Age (years)		26.1±8.6
Height (m)		1.73±0.1
Weight (kg)		72.2±17.9
BMI (kg/m^2^)		23.7±4.3
All patients (n = 33)		Count (%)
Sex	Female	20 (60.6%)
	Male	13 (39.4%)
Race	White	28 (84.8%)
	Non-White	5 (15.2%)
Ethnicity	Hispanic	3 (9.1%)
	Non-Hispanic	30 (90.9%)
Treatment	ACL Reconstruction	12 (36.4%)
	ACL Reconstruction, medial meniscus repair	8 (24.2%)
	ACL Reconstruction, lateral meniscus repair	2 (6.1%)
	ACL Reconstruction, medial and lateral meniscus repair	7 (21.2%)
	ACL Reconstruction, medial meniscus repair, partial lateral menisectomy	3 (9.1%)
	ACL Reconstruction, lateral menisectomy	1 (3.0%)

The mean implanted IAL-constructed QT autograft measured 62.3±3 .5 mm in length 9.4±0.7 mm in diameter (Table 3). Men had significantly longer graft lengths than women (p=0.021), with a trend toward larger graft diameter in males that did not reach statistical significance (p=0.0529).

**Table 3:** Implanted Quadriceps Tendon IAL Autograft Dimensions

All patients (n=33)		Females (n = 20)	Males (n = 13)	p-value
Length (mm)	62.3±3.5	61.2±3.2	64±3.3	0.021
Diameter (mm)	9.4±0.7	9.2±0.7	9.7±0.6	0.0529

Completion rates for PROMIS PI and PF questionnaires were 90.9% at baseline, 97.0% at three and six months, 75.8% at one year and 60.6% at two years in all patients. Compliance for KOOS, ADL, and Sport subscales reached 66.7%, 84.8%, 72.7%, 60.6%, and 39.4% at each time point, respectively. These declining follow-up rates over time introduce the potential for attrition bias, particularly at later time points, which should be considered when interpreting longitudinal patient-reported outcomes. PROM scores for all patients at each follow-up time point are summarized in Table 4.

**Table 4:** Patient Reported Outcome Measures (PROMs) Over Time PROMIS: Patient Reported Outcomes Measurement Information System; KOOS: Knee Injury and Osteoarthritis Index Score.

Timepoint	Baseline	3 Months	6 Months	12 Months	24 Months
PROMIS Pain Interference	61.1±6.4	54.7±9.0	54.4±8.0	48.0±8.8	46.2±8.1
PROMIS Physical Function	38.0±7.7	43.2±5.5	46.3±5.5	55.4±10.8	58.3±10.1
KOOS ADL	66.0±21.8	77.5±20.7	89.2±12.7	95.8±6.6	98.0±3.5
KOOS Sport	22.0±25.4	34.8±26.8	55.4±22.2	78.7±20.2	88.5±12.4

**Figure 6:**
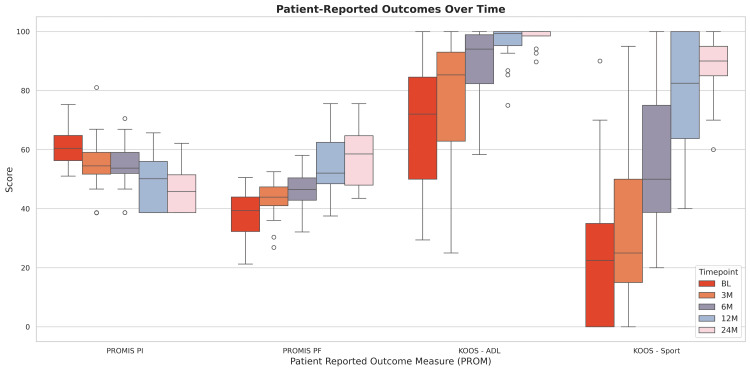
Distribution of Patient-Reported Outcome Scores Over Time Distribution of patient-reported outcome scores, including PROMIS pain interference, PROMIS physical function, KOOS activities of daily living, and KOOS sport at baseline, three, six, 12, and 24 months postoperatively in all patients. PROMIS: Patient Reported Outcomes Measurement Information System; KOOS: Knee Injury and Osteoarthritis Index Score.

All patients demonstrated significant improvements in PROMIS PI, PROMIS PF, and KOOS ADL scores from baseline to all postoperative timepoints (p<0.002, Figure 6). KOOS Sport scores improved significantly from baseline to six, 12, and 24 months (p<0.0001), with a near-significant trend toward improvement at three months (p=0.057).

**Figure 7:**
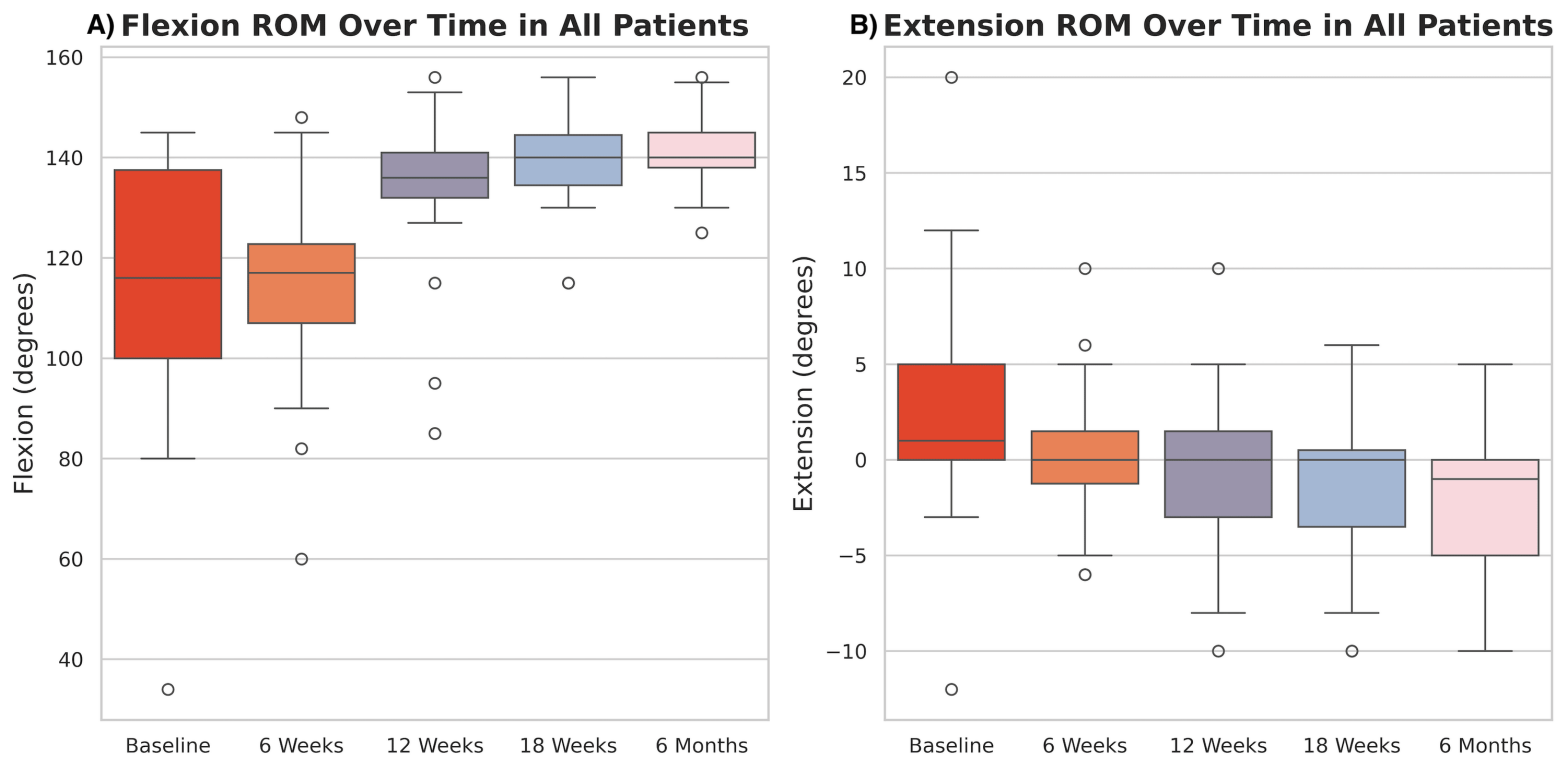
Distribution of Knee Range of Motion Over Time Boxplots visualizing distribution of knee range of motion (ROM) in flexion (left) and extension (right) in all patients at each time point.

Knee flexion and extension ROM data were available for 93.9% of patients at baseline, 97.0% at six weeks, 93.9% at 12 and 18 weeks and 100% at six months. Mean flexion and extension values are summarized in Table 5. Knee extension significantly improved at all postoperative timepoints compared to baseline (p<0.001, Figure 7). Knee flexion significantly increased in all patients at 12 weeks, 18 weeks, and six months postoperatively relative to baseline across all patients (p<0.0001), but not at six weeks (p=0.318).

**Table 5:** Mean Flexion and Extension Range of Motion Over Time

Timepoint	Baseline	6 Weeks	12 Weeks	18 Weeks	6 Months
Flexion (°)	116.2±24.7	113.9±19.3	134.6±14.5	138.1±10.4	141.6±6.8
Extension (°)	2.8±5.6	0.3±3.5	-0.5±4.4	-1.3±4.0	-2.2±3.6

**Figure 8:**
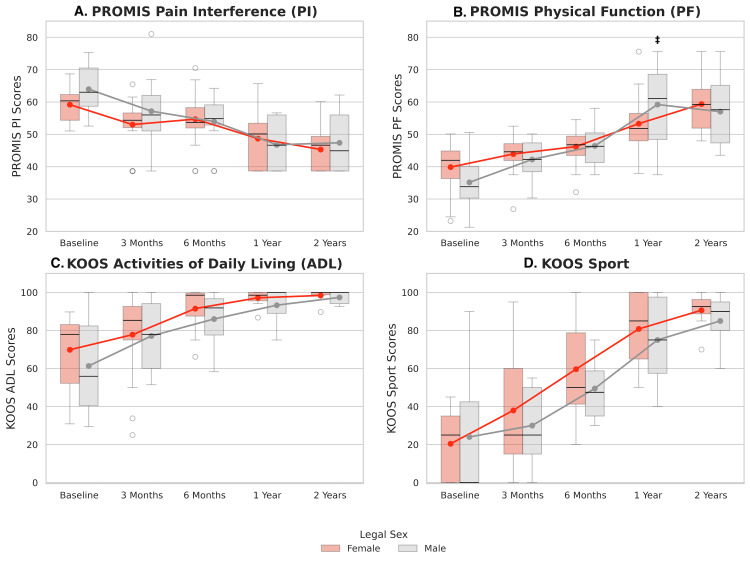
Distribution of PROMIS and KOOS Scores by Sex Boxplots show the median and interquartile range for PROMIS Pain Interference (A), PROMIS Physical Function (B), KOOS Activities of Daily Living (C), and KOOS Sport (D). Overlaid line plots represent mean scores and trajectories over time. Group comparisons for means was evaluated with independent t-tests and change from baseline was analyzed used linear mixed models. Significance was set to a=0.05. * indicates significant differences in mean scores between males and females ‡ indicates significant difference in the change in PROM score from baseline to a given timepoint between sexes. PROMIS: Patient Reported Outcomes Measurement Information System; KOOS: Knee Injury and Osteoarthritis Index Score.

For secondary analyses, patients were stratified by sex and meniscus treatment (See Appendices, Tables 6-8). There were no significant differences in mean PROM scores between men and women at any time point (p>0.05, Figure 8). LMMs indicated that sex did not significantly affect the relationship between time and PROMIS PI, KOOS ADL, or KOOS Sport scores (p>0.1, Figure 8). Men showed greater improvement in PROMIS PF at one year (β=8.1, 95% CI: (1.4, 14.8), p=0.018) compared to women; however, this difference was no longer present at two years (β=0.3, 95% CI: (-6.7, 7.4), p=0.927, Figure 8). Meniscus treatment had no significant effect on mean PROM scores or on changes in PROM scores over time (p>0.05).

**Figure 9:**
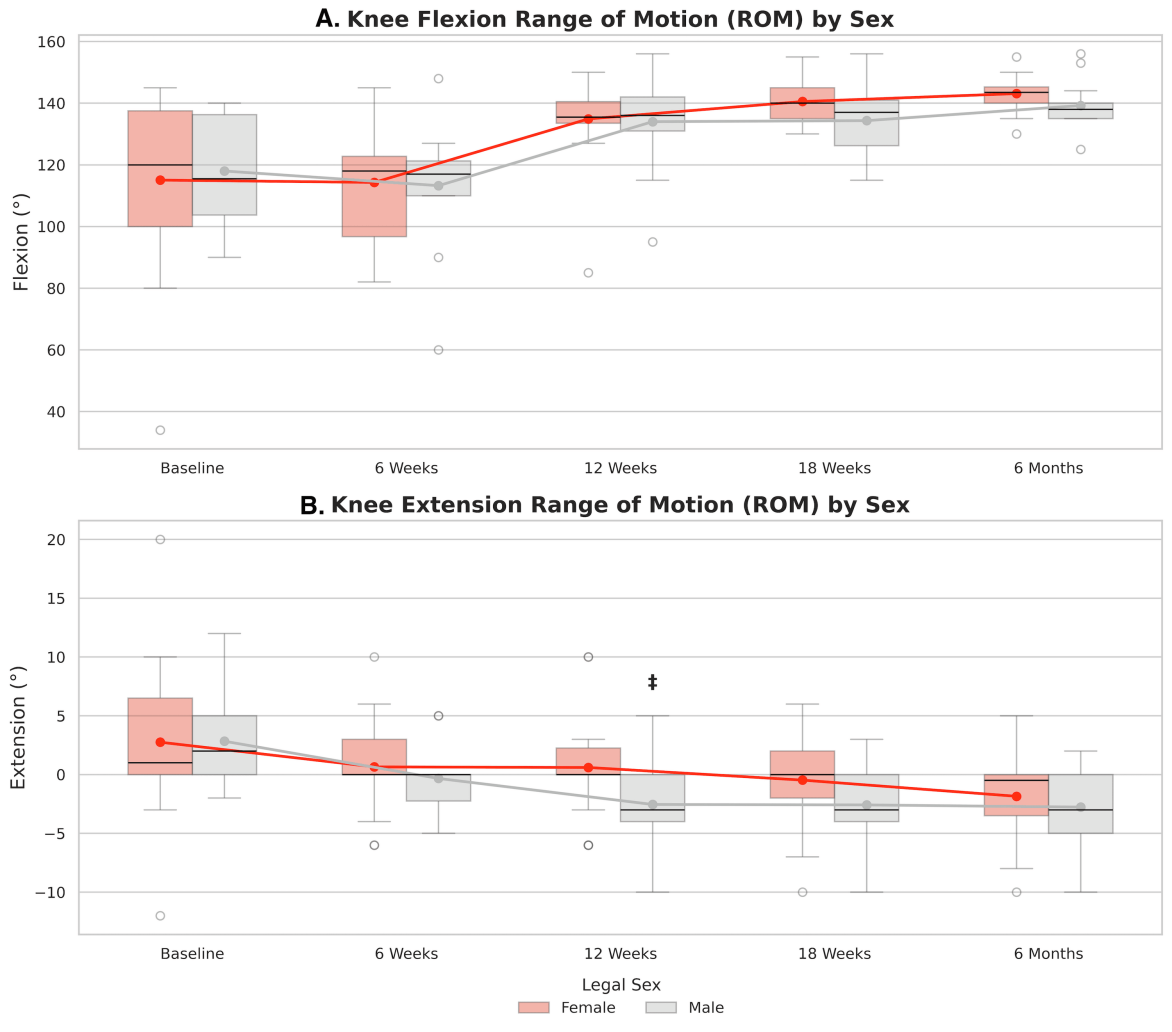
Distribution of Knee Range of Motion by Sex. Boxplots show the median and interquartile range for knee flexion (A), and knee extension (B). Overlaid line plots represent mean scores and trajectories over time. Mean group differences were evaluated using independent t-tests and differences in trajectory from baseline was analyzed using linear mixed models. Significance was set to a = 0.05. * indicates significant differences in mean ROM between males and females. ‡ indicates significant difference in the change in ROM from baseline to a given timepoint between sexes.

Mean knee flexion and extension did not significantly differ between sexes at any time point (p>0.05, Figure 9). Additionally, LMM revealed that sex did not significantly affect the relationship between knee flexion and time (p>0.05, Figure 9). Men exhibited significantly greater improvement in knee extension at 12 weeks compared to women (=-3.2°, 95% CI: (-6.2,-0.2), p=0.036, Figure 9).

**Figure 10:**
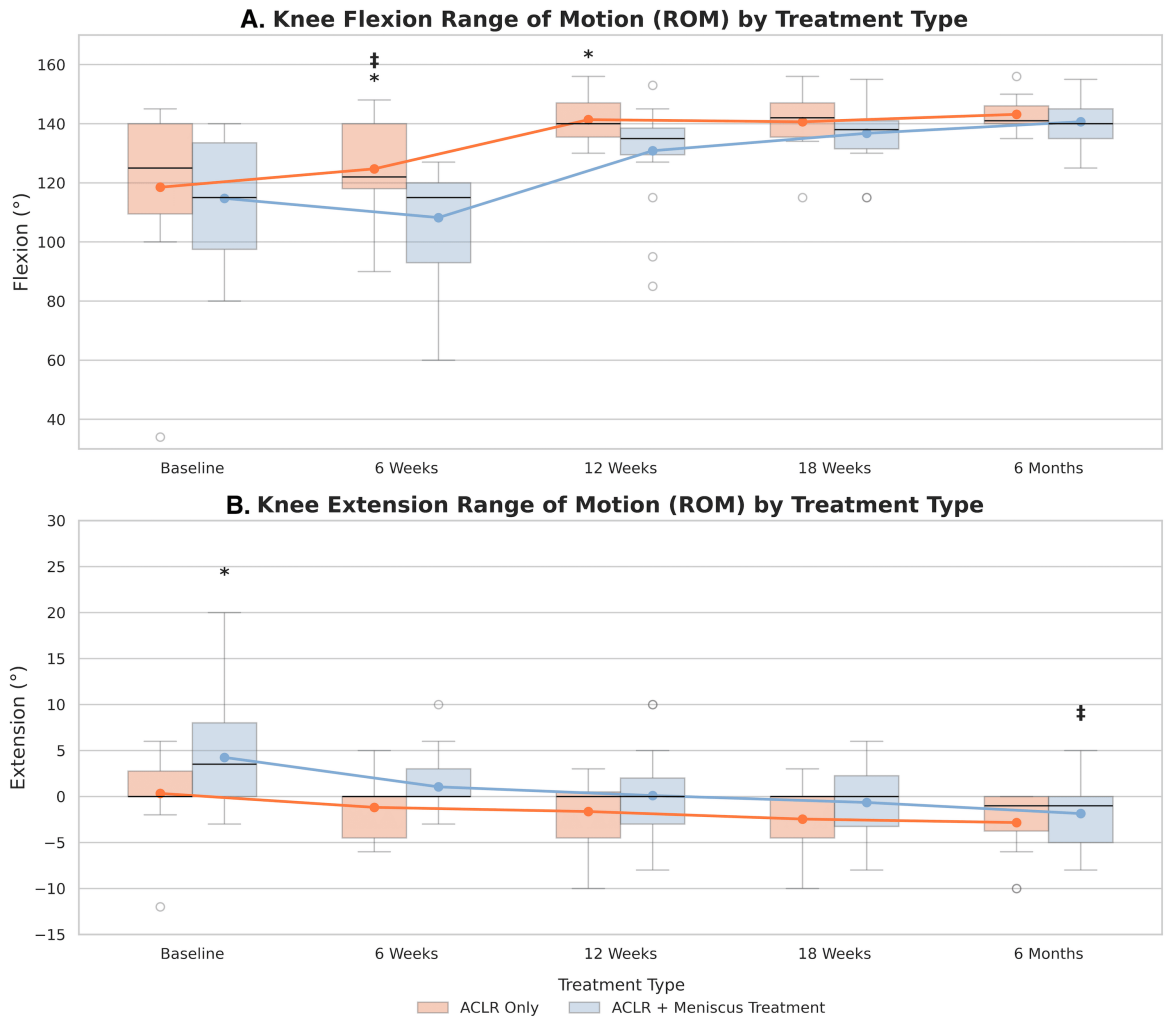
Distribution of Knee Range of Motion (ROM) Over Time by Treatment Type Boxplots show the median and interquartile range for knee flexion (A), and knee extension (B). Overlaid line plots represent mean scores and trajectories over time. Mean group differences were evaluated using independent t-tests and differences in trajectory from baseline was analyzed using linear mixed models. Significance was set to a=0.05. * indicates significant differences in mean ROM between treatment types. ‡ indicates significant difference in the change in ROM from baseline to a given timepoint between treatment types.

Patients with concomitant meniscus treatment had significantly greater knee extension at baseline (p=0.0444) but exhibited less knee flexion at six weeks (p=0.0258) and 12 weeks (p=0.0210) compared to patients who underwent ACLR only (Figure 10). They also demonstrated significantly smaller improvements in knee flexion from baseline to six weeks (=-12.8, 95% CI: (-25.1, 0.5), p=0.042) compared to the ACLR only group; however, this difference was not observed at subsequent timepoints (p>0.3, Figure 10). Additionally, meniscus treatment was associated with greater improvements in knee extension at six months relative to baseline (=-2.9, 95% CI: (-5.9, -0.05), p=0.046) compared to ACLR-only patients (Figure 10).

## Discussion

This study demonstrates that the IAL QT autograft construct has promising biomechanics performance is associated with reliable clinical outcomes following primary ACLR. The construct exhibited low cyclic elongation, especially during the second half of cyclic testing (cycles 1000-2000), and a mean ULTF exceeding 1250 N. The results are comparable to previously reported graft options and suggest adequate resistance to early stretching and structural compromise. Additionally, none of the grafts failed at the suture-tendon interface. Two patients in the retrospective case series underwent revision ACLR and both surgeries were due to subsequent injury rather than graft insufficiency. Patients demonstrated statistically significant improvements in pain, physical function, and knee-related quality-of-life outcomes over the two-year follow-up period. Improvements in self-reported outcomes were accompanied by progressive recovery of knee range of motion. Sex and meniscus treatment did not meaningfully alter outcome trajectories or final range of motion outcomes, indicating the IAL construct performs consistently across patient subgroups. Collectively, these findings support the IAL autograft as a robust graft option with both mechanical integrity and promising clinical results.

The present finding of 4.16±​0.42 mm elongation after 2000 cycles is most similar to previous values reported by Kamada et al. for QT where grafts were cyclically loaded at 50-100 N loads for 500 cycles [22]. This observed elongation may reflect a combination of early viscoelastic creep behavior and construct stabilization as initial fiber un-crimping and suture-tissue settling occur during early cycles, followed by a relative plateau as the construct reaches mechanical equilibrium. Hart et al. and Gibbs et al reported lower cyclic elongation values of 1.0 mm with 50-800 N loading for 200 cycles and 2.0±0.4 mm with 80-250 N loading for 100 cycles, respectively [8,19]. The substantial variability in loading magnitudes, cycle counts, and testing protocols across studies limits direct comparison.

The ULTF observed in the present study (1251.4±134.8 N) is most comparable to findings reported by Strauss et al., who demonstrated a mean ULTF of 1260± ​​​264 N for full-thickness QT grafts without bone blocks [11]. In several prior studies, evaluation QT grafts prepared with a whipstitch technique have been reported to have ULTF values exceeding 2000 N [8,9,25]. However, these studies employed cryogenic clamps to secure graft constructs to the testing apparatus, a method that enhances grip strength and reduces slippage [26]. This approach may contribute to the elevated loads and limits comparability of these results to the present study findings.

The most common modes of failure previously reported with suspensory fixation include suture breakage and failure at the suture tendon interface [18,19,22]. The interlinked grafts examined in this study underwent failure via sliding of the internal loop mechanism, or suture-loop breakage at forces over 1000 N. Notably, no failures were noted at the suture-cerclage interface or via suture pull-through as previously described [14,22]. The authors attribute this to interlinking of the suture cerclage with the graft, dispersing the tension force along the graft in a more uniform distribution and evening load between the suture and graft itself.

Graft failure is estimated to occur in 5% of primary ACLR cases within five years postoperatively [27]. Although nine of 33 patients in the present cohort underwent secondary procedures, none were attributed to graft failure. Arthrofibrosis similarly affects an estimated 5% of patients following ACLR with risk heightened among individuals receiving QT autografts, undergoing concomitant meniscus treatment, and who are younger, female, or were injured through contact [28-30]. The elevated rate of arthrofibrosis observed in this cohort may reflect the higher proportion of patients with these risk factors.

All patients in the clinical cohort demonstrated statistically significant improvements in KOOS ADL, KOOS Sport, PROMIS pain interference, and PROMIS physical function scores compared to baseline. Patient Acceptable Symptom States (PASS) thresholds were surpassed in all patients at initially six months and sustained throughout the follow-up period [31,32]. At one-year postoperative period, patients in the present study achieved slightly higher mean scores on KOOS ADL than those previously reported by Samuelsson et al. [33] and Kong et al. [34]. PROMIS pain interference and physical function scores were more similar to previously reported values in patients undergoing primary ACLR at one and two years postoperatively [35,36]. These findings demonstrate that this novel graft construct yields clinically meaningful improvements in function and pain and supports its effectiveness in clinical practice. Improvements in outcomes were observed alongside greater ranges of knee flexion and extension where the values observed in the present study are similar to those previously described in the literature [37]. Further, the lack of clinically meaningful differences between sex and concomitant meniscus treatment groups further highlights the versatility of this graft and supports its use across patient cohorts.

Limitations

This study has several limitations. The biomechanical analysis was conducted on a small cohort of cadaveric grafts in an ex-vivo setting, with rigid fixation of the grafts to the biomechanical testing apparatus. The linear line of pull during testing is not reflective of the physiologic vector of force or strain seen in vivo, which may limit the translational applicability of these findings, as in vivo grafts are subjected to complex, multi-planar loading conditions that may influence deformation and failure behavior. In the present study, the suture constructs were attached to the testing apparatus directly to isolate the graft specimens and did not include button fixation on the femur or tibia as is used for suspensory fixation in vivo. Further, the clinical analysis reflects a relatively small cohort from a single surgeon, which may limit generalizability to broader patient populations and introduce surgeon bias. Additionally, follow-up duration was limited to two years and compliance varied among individual patients, which may introduce attrition bias. A comparison group was not included using alternative QT fixation techniques or graft choices, restricting the ability to draw direct comparisons and limiting causal inference regarding observed biomechanics and clinical outcomes.

Future research should include prospective, comparative clinical trials evaluating the interlinked adjustable-loop construct against established fixation methods and graft types. Longitudinal studies with standardized rehabilitation metrics and return to sport outcomes also warrant further investigation. Further investigation into graft elongation under physiologic loading patterns as well as imaging-based assessments of graft maturation would provide insight into graft performance in normal physiologic conditions.

## Conclusions

This study evaluated the biomechanical performance and clinical outcomes of a novel IAL suspensory fixation technique for all-soft tissue quadriceps tendon autografts used in ACL reconstruction. Biomechanical testing demonstrated low cyclic elongation and high ultimate load to failure with no failures at the suture-tendon interface, suggesting the interlinked design may enhance load distribution and mitigate common soft tissue failure modes. Patients treated with this construct showed significant improvement in knee pain, function, and range of motion with no graft failures. Clinical outcomes were not meaningfully influenced by sex or concomitant meniscal procedures. These findings support the IAL technique as a biomechanically robust and clinically effective method for preparing quadriceps tendon autografts for ACL reconstruction. Future prospective, comparative studies are warranted to confirm these findings and determine if this construct offers advantages over traditional QT fixation techniques.

## Appendices

**Table 6:** PROMs by Sex and Meniscus Treatment PROMIS: Patient Reported Outcomes Measurement Information System; KOOS: Knee Injury and Osteoarthritis Index Score; ACLR: anterior cruciate ligament reconstruction.

All Patients (n=33)	Timepoint	Baseline	3 Months	6 Months	12 Months	24 Months
PROMIS Pain Interference	Females	59.2±5.3	53.0±7.3	54.7±8.1	48.7±9.2	45.3±7.2
	Males	64.0±7.0	57.1±10.9	53.9±8.1	46.8±8.3	47.4±9.4
	ACLR Only	58.8±5.8	53.0±7.9	54.3±8.8	49.9±10.6	45.9±8.9
	ACLR+Meniscus Treatment	62.4±6.5	55.7±9.7	54.5±7.7	46.7±7.7	46.7±7.4
PROMIS Physical Function	Females	39.8±7.3	43.9±5.4	46.2±5.1	53.3±9.3	59.4±9.0
	Males	35.1±7.6	42.2±5.8	46.4±6.4	59.2±12.8	57.0±11.8
	ACLR Only	39.8±5.7	44.6±4.7	45.8±5.7	52.6±12.2	60.2±70.8
	ACLR+Meniscus Treatment	36.9±8.6	42.4±5.9	46.6±5.5	57.3±9.8	57.5±10.1
KOOS ADL	Females	69.8±19.1	77.8±2.3	91.5±11.1	97.2±3.8	98.4±3.5
	Males	61.3±24.8	77.1±19.1	86.0±14.7	93.3±9.8	97.3±3.7
	ACLR Only	71.7±20.1	79.6±15.5	89.6±13.7	97.0±5.0	99.7±0.7
	ACLR+Meniscus Treatment	62.0±22.8	76.1±23.8	88.9±12.6	95.0±7.5	96.9±4.1
KOOS Sport	Females	20.4±16.4	37.9±30.5	59.6±25.0	80.8±18.2	90.6±9.8
	Males	24.0±24.1	30.0±20.1	49.5±16.9	75.0±24.5	85.0±15.8
	ACLR Only	17.2±17.5	29.5±28.0	52.2±15.6	80.0±22.8	93.0±4.5
	ACLR+Meniscus Treatment	25.3±29.9	38.2±26.2	57.3±25.6	77.9±19.2	85.6±14.7

**Table 7:** Knee Flexion and Extension ROM by Sex and Meniscus Treatment ACLR: anterior cruciate ligament reconstruction; ROM: range of motion.

All Patients (n = 33)	Timepoint	Baseline	6 Weeks	12 Weeks	18 Weeks	6 Months
							Flexion (° )	Females	115.0±6.5	114.3±18.5	134.9±13.3	140.5±6.8	143.1±5.6
Males	118.0±18.5	113.2±21.4	134.0±17.0	134.3±13.9	139.2±8.1		
ACLR Only	118.5±30.8	124.7±18.8	141.4±7.9	140.6±10.9	143.2±6.1		
ACLR + meniscus treatment	114.7±20.8	108.2±17.4	130.8±16.0	136.7±10.1	140.7±7.2		
Extension (° )	Females	2.7±6.5	0.6±3.8	0.6±4.2	-0.5±4.0	-1.8±3.6
	Males	2.8±3.9	-0.3±3.0	-2.5±4.2	-2.6±3.8	-2.8±3.7
	ACLR Only	0.3±4.6	-1.2±3.6	-1.6±4.1	-2.4±4.4	-2.8±3.8
	ACLR + meniscus treatment	4.2±5.8	1.0±3.2	0.1±4.6	-0.65±3.7	-1.8±3.5

**Table 8:** Differences in Mean PROM Scores between Timepoints PROMIS: Patient Reported Outcomes Measurement Information System; KOOS: Knee Injury and Osteoarthritis Index Score.

All Patients (n = 33)	Timepoint	Baseline to 3 Month	Baseline to 6 Months	Baseline to 1 Year	Baseline to 2 Years
PROMIS Pain Interference	Delta score	-6.5	-4.9	-10.4	-12.9
	95% CI	(-10.5, -2.5)	(8.8, -1.0)	(-14.6, -6.2)	(-17.7, -8.1)
	p-value	0.001	0.014	<0.0001	<0.0001
PROMIS Physical Function	delta score	5.3	8.2	16.9	19.3
	95% CI	(2.3, 8.3)	(5.2, 11.3)	(13.6, 20.2)	(15.7, 22.8)
	p-value	0.001	<0.0001	<0.0001	<0.0001
KOOS Activities of Daily Living	delta score	11.6	24.1	30.6	31.1
	95% CI	(4.3, 18.9)	(16.6, 31.7)	(22.6, 38.6)	(21.7, 40.4)
	p-value	0.002	<0.0001	<0.0001	<0.0001
KOOS Sport	delta score	12.1	32.9	56.5	64.7
	95% CI	(-0.3, 24.5)	(20.1, 45.7)	(43.0, 69.9)	(48.9, 80.4)
	p-value	0.057	<0.0001	<0.0001	<0.0001
